# Circ-FOXM1 promotes the proliferation, migration and EMT process of osteosarcoma cells through FOXM1-mediated Wnt pathway activation

**DOI:** 10.1186/s13018-022-03207-0

**Published:** 2022-07-07

**Authors:** Hao Zhang, Qiongqiong Zhou, Weimin Shen

**Affiliations:** 1 Department of Burn and Plastic Surgery, Children’s Hospital of Nanjing Medical University, 72 Guangzhou Road, Nanjing, 210008 Jiangsu China; 2 Department of Otolaryngology, Children’s Hospital of Nanjing Medical University, Nanjing, 210008 China

**Keywords:** Osteosarcoma, Circ-FOXM1, miR-320a, miR-320b, FOXM1

## Abstract

**Background:**

Osteosarcoma (OS) is a malignant bone tumor that commonly occurs in adolescents with a high mortality rate and frequent pulmonary metastasis. Emerging evidence has suggested that circular RNAs (circRNAs) are important regulators in multiple biological activities of carcinomas. Nevertheless, the role of circRNAs derived from forkhead box M1 (FOXM1), a well-accepted modulator of OS progression, has not been discussed in OS.

**Methods:**

Quantitative real-time polymerase chain reaction (qRT-PCR) was utilized to test circ-FOXM1 (hsa_circ_0025033) expression in OS cell lines. Cell counting kit-8 (CCK-8), 5-ethynyl-2’-deoxyuridine (EdU), terminal deoxynucleotidyl transferase dUTP nick end labeling (TUNEL), transwell assays and western blot analysis of epithelial-mesenchymal transition (EMT) markers were conducted to evaluate cell proliferation, apoptosis, migration, and EMT process. Luciferase reporter assay and RNA-binding protein immunoprecipitation (RIP) assay were utilized to detect the interaction of circ-FOXM1 and RNAs.

**Results:**

High expression of circ-FOXM1 was detected in OS cell lines. Functionally, circ-FOXM1 knockdown inhibited the proliferation, migration and EMT process, whereas induced the apoptosis of OS cells. From the aspect of molecular mechanism, circ-FOXM1 was discovered to upregulate FOXM1 expression via sponging miR-320a and miR-320b, therefore activating Wnt signaling pathway. Besides, rescue experiments elucidated that circ-FOXM1 regulated cellular activities of OS cells via FOXM1. Further, in vivo assays supported that loss of circ-FOXM1 restrained OS tumor growth.

**Conclusion:**

Circ-FOXM1 facilitated the malignant phenotypes of OS cells through FOXM1-mediated Wnt pathway activation, revealing circ-FOXM1 as a potential biomarker for OS treatment.

**Supplementary Information:**

The online version contains supplementary material available at 10.1186/s13018-022-03207-0.

## Background

Osteosarcoma (OS), the most common primary bone tumor, mainly occurs in adolescents and children, with a high mortality rate [1, 2]. In spite of the advances made in surgical removal, radiotherapy and neoadjuvant chemotherapy for OS, the prognosis still remains dismal as a result of metastatic spread [3, 4]. The 5-year survival rate of OS patients with distant metastases is about 30% [5]. Nevertheless, an in-depth understanding of molecular mechanisms of OS is vitally important.

Mounting evidence has reported that dysregulated noncoding RNAs (ncRNAs) are related to the pathogenesis, initiation and progression of OS [6–8]. Circular RNAs (circRNAs), a new type of ncRNAs with a closed loop, are highly conserved and stable due to their structure [9]. They play a crucial role in tumor development since they can regulate gene expression via diverse mechanisms, including interacting with RNA-binding proteins (RBPs), acting as sponges of microRNAs (miRNAs), and so on [10–12]. For example, hsa_circ_0001564 modulates OS cell proliferation and apoptosis via acting a miRNA sponge [13]; circRNA_001569 promotes the proliferation and invasion of colorectal cancer cells via targeting miR-145 [14]; and suppression of PABPN1 translation by circPABPN1 was relied on HuR [15]. Besides, several major signaling cascades involved in cancer development have also been identified to be the downstream of circRNAs, such as the Wnt/β-catenin, VEGF, PI3K/AKT, MAPK and Notch signaling pathways [16]. For instance, translation of circβ-catenin promotes tumor growth in liver cancer by activating the Wnt/β-catenin pathway [17].

Circ-FOXM1 (hsa_circ_0025033) is a newly recognized cancer-related circRNA located at chr12: 2966846–2983691 and derived from FOXM1 which has been report to be overexpressed in OS and promotes OS progression [18]. Presently, circ-FOXM1 has been proven to be upregulated in glioblastoma as screened by circRNA microarray [19]. Moreover, circ-FOXM1 promotes cell progression by sponging miR-1304-5p that targets PPDPF and MACC1 in non-small cell lung cancer [20]; upregulated circ-FOXM1 facilitates cell proliferation and invasion via absorbing miR-1231 and miR-1304 in papillary thyroid cancer [21]. However, the role of circ-FOXM1 in OS is not fully investigated.

MiRNAs are endogenous single stranded ncRNA molecules of 21–23 nt length [22]. They can negatively regulate their downstream target messenger RNAs (mRNAs) so as to be involved in the regulation of cancer development and progression. For example, miR-1231 expression is down-regulated in prostate cancer and it has prognostic and functional implications for this disease [23]. MiR-125 represses colorectal cancer cell proliferation and invasion via targeting TAZ [24]. MiR-223-3p inhibits OS metastasis and progression by targeting CDH6 [25].

This research aims to investigate the biological function of circ-FOXM1 in OS. Further, the relationship between circ-FOXM1 and its host gene FOXM1in OS cells was also studied. Our research may offer a potential therapeutic target for OS, shedding light on OS therapy.

## Materials and methods

### Cell culture

Human normal osteoblastic cell line hFOB1.19 and four kinds of OS cell lines (MG-63, HOS, 143B and U2OS) were acquired commercially from Chinese Academy of Sciences (Shanghai, China). All cell lines were allowed to grow in Dulbecco’s modified Eagle medium (DMEM; Invitrogen, Carlsbad, CA, USA) supplemented with 10% fetal bovine serum (FBS; HyClone, Logan, UT) at 37 °C in 5% CO_2_.

### Cell transfection plasmids

143B and U2OS cells were seeded into 6-well plates (Corning, Shanghai, China). When reaching 80–90% confluence, cells were, respectively, transfected with three kinds of short hairpin RNAs (shRNAs) specific for circ-FOXM1 (namely (sh-circ-FOXM1#1/2/3) and negative control of shRNA (sh-NC) in line with the user guide of Lipofectamine™ 3000 (Invitrogen)). The shRNAs were all constructed by GenePharma (Shanghai, China). Meanwhile, pcDNA3.1 vector (Invitrogen) loading with FOXM1 cDNA sequence (pcDNA3.1/FOXM1) was used to overexpress FOXM1, with empty vector pcDNA3.1 served as the control. To mimic or inhibit miR-320a and miR-320b, miR-320a and miR-320b mimics or inhibitors were designed by RiboBio (Guangzhou, China), along with their respective controls. After 48 h of transfection, cells were harvested for subsequent study.

### Quantitative real-time polymerase chain reaction (qRT-PCR)

Total RNA was extracted from OS cell lines using TRIzol reagent (Life Technologies Corporation, Carlsbad, CA, USA). After measuring RNA concentration, 100 μg of RNA was used for reverse transcription by using High-Capacity cDNA Reverse Transcription Kit (Applied Biosystems™, Foster City, CA, USA) or TaqMan® MicroRNA Reverse Transcription Kit (Applied Biosystems™). qRT-PCR analysis was carried out using SYBR Premix Ex Taq™ II (Takara, Osaka, Japan) and the ABI 7500 Fast Real-Time PCR System (Thermo Scientific™, Waltham, MA, USA). The Ct value of samples was recorded, and relative gene expression was calculated by the 2^−ΔΔCt^ method. U6 small nuclear RNA was seen as the internal control for miRNAs and GAPDH (glyceraldehyde-3-phosphate dehydrogenase) was seen as the internal control for other genes. All reactions were performed in triplicate.

### In vivo experiments

Nine nude mice (male, 6-week-old, 22–25 g) were acquired from Slac Laboratories (Shanghai, China). Animal study was approved by the Ethics Committee of Children’s Hospital of Nanjing Medical University. Mouse model was established by subcutaneous injection of 1 × 10^7^ sh-circ-FOXM1#1/2-transfected U2OS cells into the back flank of mice as experimental groups. The mice injected with equal amount of sh-NC-transfected U2OS cells served as control group. One week post inoculation, the size of tumors in the mice was measured every three days. Twenty-eight days later, the mice were photographed before sacrificed via cervical dislocation, and the xenografts collected from them were imaged and weighed.

### Immunohistochemical (IHC) staining and terminal deoxynucleotidyl transferase-mediated dUTP nick-end labeling (TUNEL) staining

After pretreatment with ice-cold phosphate buffer saline (PBS) and fixed in 10% buffered neutral formalin (Beijing Solaibao Technology, Beijing, China), tumor xenografts were paraffin-embedded and cut into 4-μm sections. The sections were subjected to IHC staining of Ki67 and FOXM1 expression and TUNEL staining of apoptosis in xenografts after being deparaffinized and rehydrated as described [26]. For IHC analysis, antigen retrieval was performed using boiling citrate buffer (0.01 M, pH 6.0; Dako, Copenhagen, Denmark), and endogenous peroxidase activity was blocked via 0.3% H_2_O_2_. Then, the sections were subjected to incubation with the primary antibody against Ki67 (anti-Ki67; #9449, 1:500 dilution, Cell signaling Technology, Boston, MA, USA) or FOXM1 (anti-FOXM1; #20459, 1:600 dilution, Cell signaling Technology) at 4 °C overnight. After PBS washing, the sections were incubated with secondary antibodies (ab205718, 1:1500 dilution, Abcam, Cambridge, MA, USA) for 1 h at room temperature. After PBS washing, the sections were dyed by diaminobenzidine (DAB, shown in brown). For TUNEL staining, cell apoptosis in above sections was tested by use of In Situ Cell Death Detection Kit (Roche, Mannheim, Germany) as per the manufacturer’s instructions. Images were acquired by a light microscope (Olympus, Tokyo, Japan).

### Cell proliferative assays

Cell counting kit-8 (CCK-8) and 5-ethynyl-2’-deoxyuridine (EdU) assays were used to analyze OS cell proliferation. For CCK-8 assay, 143B and U2OS cells were put into 96-well plates (Corning) for 24, 48 or 72 h of incubation. Then, each well of the plates was processed with 10 μL of CCK-8 solution (Beyotime, Beijing, China) for 2 h. The cellular absorbance was detected by a microplate reader (Molecular Devices, Carlsbad, CA, USA) at 450 nm. For EdU incorporation assay, the experiments were performed by the use of EdU detection kit from RiboBio. Briefly, cells were incubated with 100 μL of 50 μM EdU medium diluent in culture plates for 3 h. After fixation, cells were treated with 100 μL of the 0.5% Troxin X-100 and 100 μL of 1 × Apollo® 488 fluorescent staining reaction liquid for 30 min at 37 °C. Cell nuclei were dyed with DAPI (Sigma-Aldrich, St. Louis, MO, USA). All proliferative assays were repeated for at least three times.

### Cell apoptosis assay

Apoptotic cells were detected via TUNEL assay by use of In Situ Cell Death Detection Kit (Roche). The transfected cell lines were reaped and rinsed in PBS for 5 min and then subjected to 0.1% TritonX-100 (Sigma-Aldrich) in 1% sodium citrate (Supelco, Bellefonte, PA, USA) on ice for 2 min. Following labeling with the fluorescein-TUNEL reagent at 37 °C for 1 h, DAPI was used to stain cell nucleus. The final images of three different experiments were visualized under an inverted microscope (Olympus).

### Cell migration assay

After transfection, cells were trypsinized and adjusted to 2 × 10^5^ cells/mL, followed by incubation in the upper chamber of a transwell (Costar, Lowell, MA) with 8 μm pores. The lower chamber was supplied with 500 μL of culture medium with 10% FBS. One day later, a cotton swab was used to scrape off the non-migrating cells in the upper chamber. The cells which filtered to the other side of the membrane were dyed with 2% crystal violet (Sigma-Aldrich) and counted under an inverted microscope (Olympus) at × 200 magnification. Experiment was performed in triplicate.

### Western blot analysis

Cell lines were lysed in radio immunoprecipitation assay (RIPA) protein extraction reagent (Beyotime). Protein concentration was detected by BCA Protein Assay kit (Thermo Fisher Scientific, Rockford, IL). Samples were separated on sodium dodecyl sulfate–polyacrylamide gel electrophoresis (SDS-PAGE), transferred onto nitrocellulose (NC) membranes (Whatman, Little Chalfont, UK) and blocked in TBST with 5% nonfat milk for 2 h. Protein was incubated with the primary antibodies overnight at 4 °C and washed thrice in PBS, followed by incubation with secondary antibodies (ab205718 or ab6789, 1:10000 dilution, Abcam). Anti-GAPDH antibody (ab8245, 1:10000 dilution, Abcam) served as an endogenous reference. The signals were detected using the ECL Plus Detection Kit (Pierce, Rockford, IL). Each procedure was performed for more than twice. Primary antibodies were as follows: anti-MMP2 (ab97779, 1:2000 dilution, Abcam), anti-MMP9 (ab76003, 1:5000 dilution, Abcam), anti-E-cadherin (ab1416, 1:50 dilution, Abcam), anti-N-cadherin (ab98952, 1:2000 dilution, Abcam), anti-FOXM1 (#20459, 1:1000 dilution, Cell signaling Technology), anti-β-catenin (ab32572, 1:5000 dilution, Abcam), anti-C-myc (#18583, 1:1000 dilution, Cell signaling Technology).

### Immunofluorescence (IF) staining assay

After transfection, cells on coverslips were subjected to fixation with 4% paraformaldehyde, and then blocked with 5% BSA. After that, cells were cultivated with primary antibodies against β-catenin (ab32572, 1:250 dilution, Abcam) at 4 °C for one night, and with fluorescence-conjugated secondary antibodies (ab150080, 1:200 dilution, Abcam) at room temperature for 2 h next. DAPI was utilized to stain the nuclei. Finally, the fluorescence microscope (Leica, Wetzlar, Germany) was utilized to acquire images. The experiment was repeated at least three times.

### Luciferase reporter assay

FOXM1 promoter was amplified by PCR and cloned into pGL3-Basic vector (Promega Corporation, Madison, WI, USA). U2OS and 143B cells were seeded on 96-well plates (8 × 10^3^/well) and co-transfected with luciferase reporter plasmids containing FOXM1 promoter and sh-circ-FOXM1 or sh-NC. 48 h later, luciferase activity was measured using the Dual-Luciferase Reporter Assay System (Promega) in accordance with the user guide. For analyzing the interaction between miR-320a/miR-320b and circ-FOXM1 or FOXM1, U2OS and 143B cells were co-transfected with psiCHECK-2 vector (Promega) containing circ-FOXM1-WT/MUT or FOXM1-WT/MUT, and miR-320a/miR-320b mimics or miR-NC. Experimental data were obtained from three independent replicates.

### RNA-binding protein immunoprecipitation (RIP) assay

RIP assay was conducted using Magna RIP RNA-Binding Protein Immunoprecipitation Kit (Millipore, Billerica, MA, USA) following the supplier’s protocol. U2OS and 143B cells were rinsed in PBS and subjected to RIP buffer at 4 °C for 30 min, and incubated with protein A/G sepharose beads conjugated to antibodies against Ago2 (66720–1-Ig, 1:200 dilution, Proteintech, Chicago, IL, USA) or normal mouse immunoglobulin G (IgG; A0408, 1:200 dilution, Beyotime). At length, immunoprecipitated RNA from cell lysates of three different experiments was extracted for qRT-PCR analysis.

### Statistical analysis

All experimental results were expressed as the mean ± SD, and each experiment was repeated in triplicate. Statistical analyses and graphical depictions were conducted using GraphPad Prism 5.0 (GraphPad, San Diego, CA, USA). Student’s t test or one-way analysis of variance (ANOVA) was used to evaluate the differences between results. Statistical significance was set as *P < 0.05, **P < 0.01.

## Results

### Circ-FOXM1 was overexpressed in OS cells

**Fig. 1:**
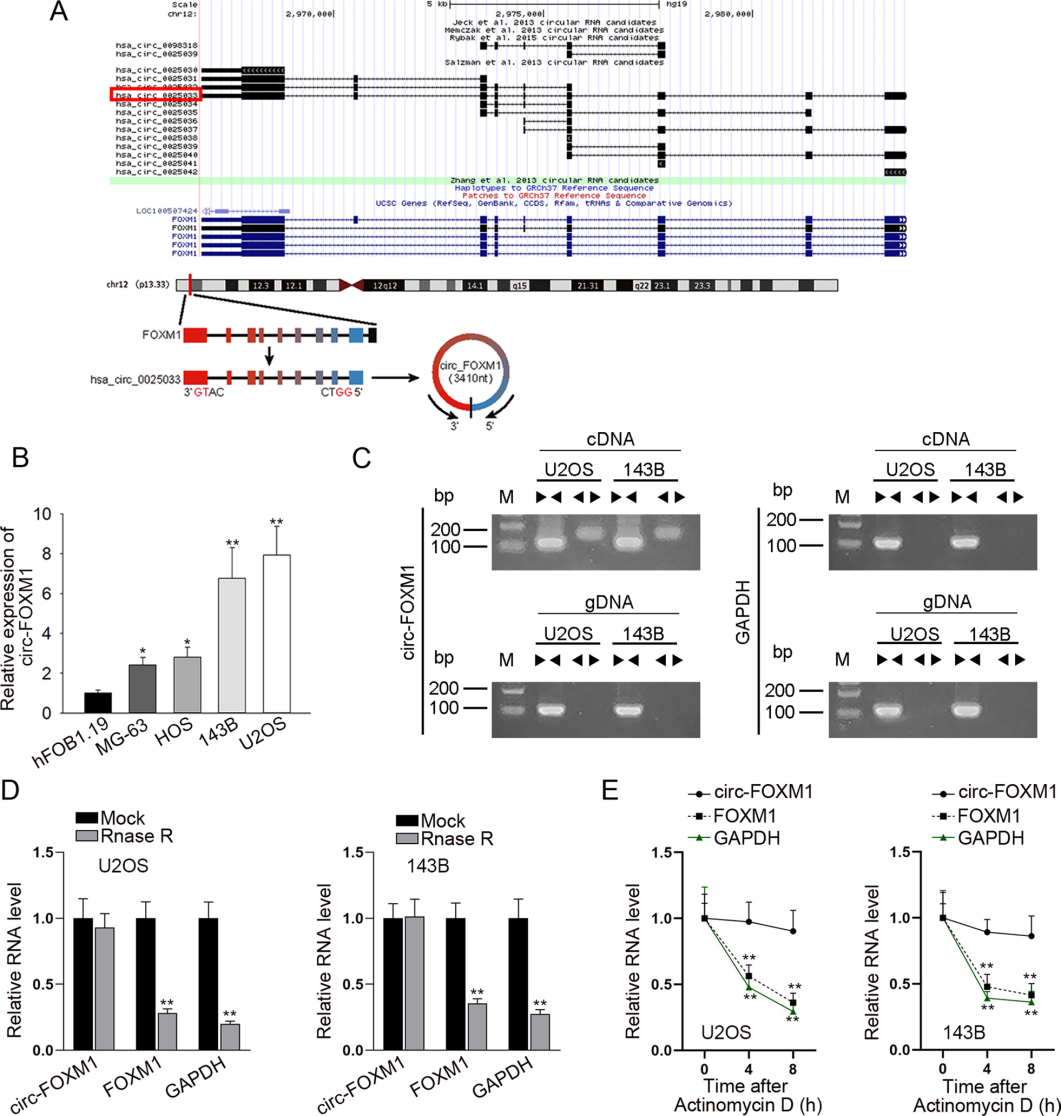
Circ-FOXM1 was overexpressed in OS cells. **a** The genomic loci of circ-FOXM1. **b** The overexpression of circ-FOXM1 in OS cells (MG-63, HOS, 143B and U2OS) compared to normal osteoblast hFOB1.19 cells, as tested by qRT-PCR. **c–e** Agarose gel electrophoresis, RNase R treatment and Actinomycin D treatment were used to examine the difference between circ-FOXM1 and linear FOXM1 mRNA. *P < 0.05, **P < 0.01

First of all, through UCSC (http://genome.ucsc.edu/) database, we found that hsa_circ_0025033, a circRNA derived from FOXM1 (subsequently named circ-FOXM1), was located in chr12: 2966846–2983691 and had a length of 3410 bp in size (Fig. 1a). Following, according to the results from qRT-PCR, circ-FOXM1 expression was upregulated in OS cell lines (MG-63, HOS, 143B and U2OS), compared with that in human osteoblast hFOB1.19 cell line (Fig. 1b). 143B and U2OS cell lines displayed a relatively higher expression of circ-FOXM1 among four OS cell lines. Thus, we selected these two cells for the subsequent assays. Then, data from agarose gel electrophoresis verified the circular characteristic of circ-FOXM1 (Fig. 1c). Subsequently, it was showed that, after RNase R treatment, FOXM1 expression level was significantly reduced, whereas circ-FOXM1 was resistant to RNase R digestion (Fig. 1d). Besides, we discovered that in face of Actinomycin D treatment, the RNA level of circ-FOXM1 almost unchanged while that of FOXM1 was significantly declined (Fig. 1e). Above data validated the closed loop structure of circ-FOXM1. Collectively, circ-FOXM1 was expressed at high level in OS cells.

### Knockdown of circ-FOXM1 suppressed cell proliferation, migration and EMT process in OS

**Fig. 2:**
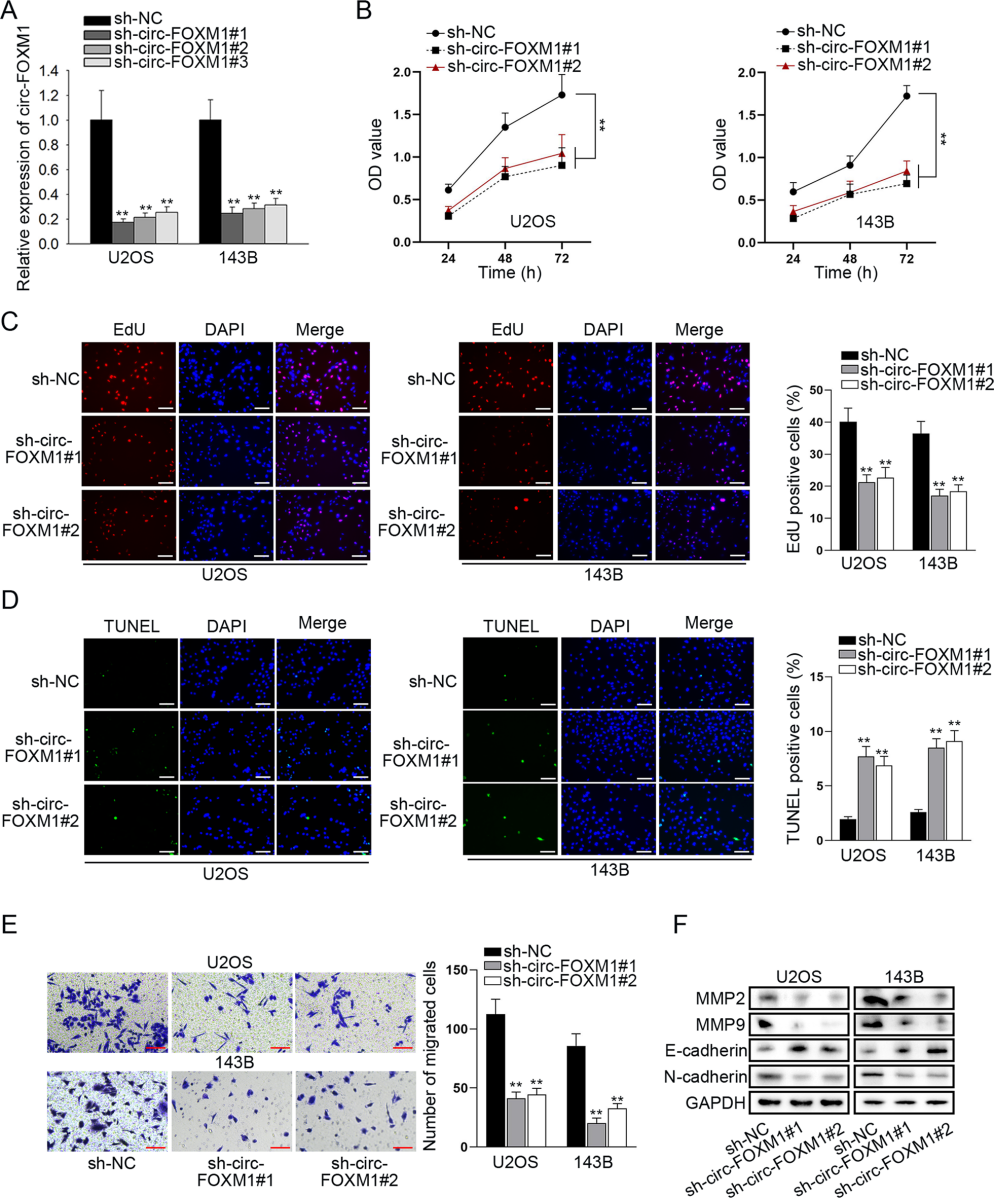
Knockdown of circ-FOXM1 suppressed OS cell proliferation, migration and EMT process. **a** The inference efficacy of sh-circ-FOXM1#1/2/3 in two OS cells was determined by qRT-PCR. **b, c** Cell proliferation was measured through CCK-8 and EdU (bar value = 50 μm) assays in sh-circ-FOXM1-transfected cells. **d** Cell apoptosis was estimated by the use of TUNEL (bar value = 50 μm) assay in sh-circ-FOXM1-transfected cells. **e** Transwell (bar value = 50 μm) assay was performed to evaluate cell migration in sh-circ-FOXM1-transfected cells. **f** The levels of EMT-associated proteins were measured using western blot in sh-circ-FOXM1-transfected cells. **P < 0.01

To explore the functional role of circ-FOXM1 in OS, we transfected shRNAs specifically targeting circ-FOXM1 (sh-circ-FOXM1#1/2/3) into two OS cells. The expression level of circ-FOXM1 was markedly down-regulated in U2OS and 143B cells after transfection of sh-circ-FOXM1 plasmids, and sh-circ-FOXM1#1 and sh-circ-FOXM1#2 exhibited the better knockdown efficiency (Fig. 2a). Next, functional assays were performed to examine the effects of circ-FOXM1 interference on OS cells. CCK-8 assay demonstrated that OS cell viability was significantly inhibited by knockdown of circ-FOXM1 (Fig. 2b). EdU assay results showed that the ratio of EdU positive cells was obviously declined in circ-FOXM1-inhibited groups (Fig. 2c), suggesting that cell proliferation was inhibited by circ-FOXM1 depletion. In addition, TUNEL assay data showed that circ-FOXM1 silencing dramatically decreased the number of TUNEL positive cells (Fig. 2d), which indicated that circ-FOXM1 depletion induces OS cell apoptosis. As evidenced by transwell assay, the number of migrated cells was distinctly reduced when circ-FOXM1 was silenced (Fig. 2e), which illustrated that OS cell migration could be restrained by knockdown of circ-FOXM1. It was also observed from western blot analyses that the protein levels of MMP2 and MMP9 were relatively reduced by circ-FOXM1 depletion. Meanwhile, E-cadherin (an epithelial marker) protein expression was increased and N-cadherin (a mesenchymal marker) protein expression was lessened after the down-regulation of circ-FOXM1 (Fig. 2f). The data suggested that OS cell migration and EMT process could be repressed by silencing of circ-FOXM1. Taken together, circ-FOXM1 suppression inhibited the proliferation, migration and EMT process of OS cells.

### Circ-FOXM1 could regulate the expression level of FOXM1 post-transcriptionally

**Fig. 3:**
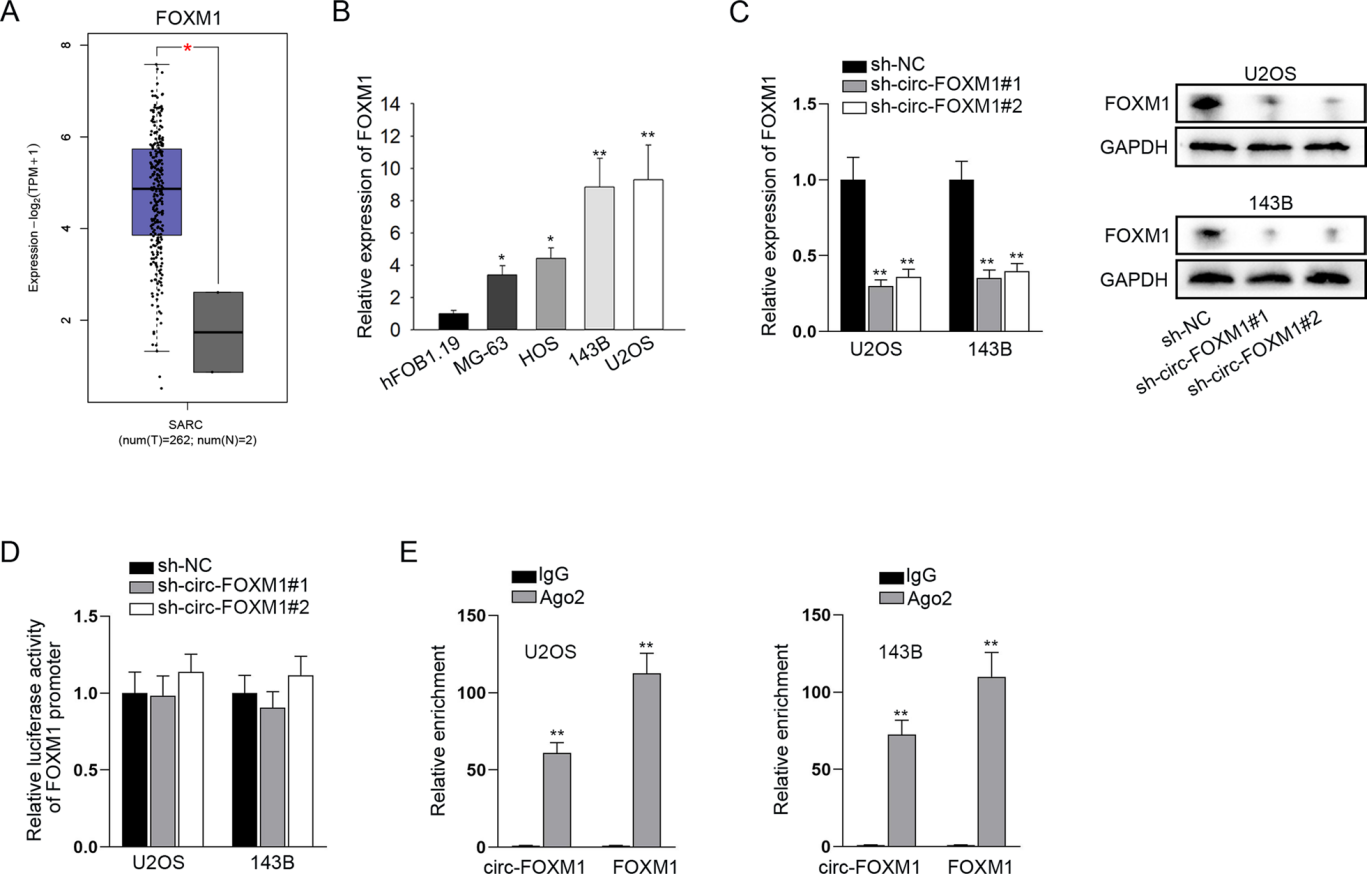
Circ-FOXM1 could regulate the expression level of FOXM1 post-transcriptionally. **a** From GEPIA database, we found the upregulation of FOXM1 in sarcoma (SARC) tissues. **b** qRT-PCR examined the relatively high expression of FOXM1 in OS cells, in comparison with normal hFOB1.19 cells. **c** The mRNA expression and protein level of FOXM1 were detected in cells transfected with sh-NC or sh-circ-FOXM1#1/2 through qRT-PCR and western blot analyses. **d** Luciferase reporter assay detected the impact of circ-FOXM1 knockdown on FOXM1 transcription. **e** RIP assay confirmed the coexistence of circ-FOXM1 and FOXM1 in Ago2-assembled RISCs. *P < 0.05, **P < 0.01

Since FOXM1 was the host gene of circ-FOXM1, we wondered whether circ-FOXM1 could influence the expression of FOXM1 in OS. Firstly, by browsing the GEPIA (http://gepia.cancer-pku.cn/) database, we discovered the overt upregulation of FOXM1 in sarcoma (SARC) tissues (Fig. 3a). Through qRT-PCR analyses, we validated the elevation of FOXM1 expression in OS cell lines versus control (Fig. 3b). Moreover, we investigated the direct impact of circ-FOXM1 on FOXM1 and found that FOXM1 mRNA expression and protein levels were largely reduced in circ-FOXM1-depleted OS cells (Fig. 3c), hinting the positive correlation between circ-FOXM1 and FOXM1. Then, luciferase reporter assay showed that circ-FOXM1 knockdown caused no change in the luciferase activity of FOXM1 promoter (Fig. 3d), excluding the potential of circ-FOXM1 to regulate FOXM1 at the transcriptional level. Further, data from RIP assay showed that circ-FOXM1 and FOXM1 were both highly enriched in the anti-Ago2-bound immunoprecipitates (Fig. 3e), which illustrated that they both existed in RNA inducing silencing complexes (RISCs). These data suggested that circ-FOXM1 might regulate FOXM1 expression at the post-transcriptional level through competing endogenous RNA (ceRNA) network.

### Circ-FOXM1 targeted miR-320a and miR-320b to modulate FOXM1

**Fig. 4:**
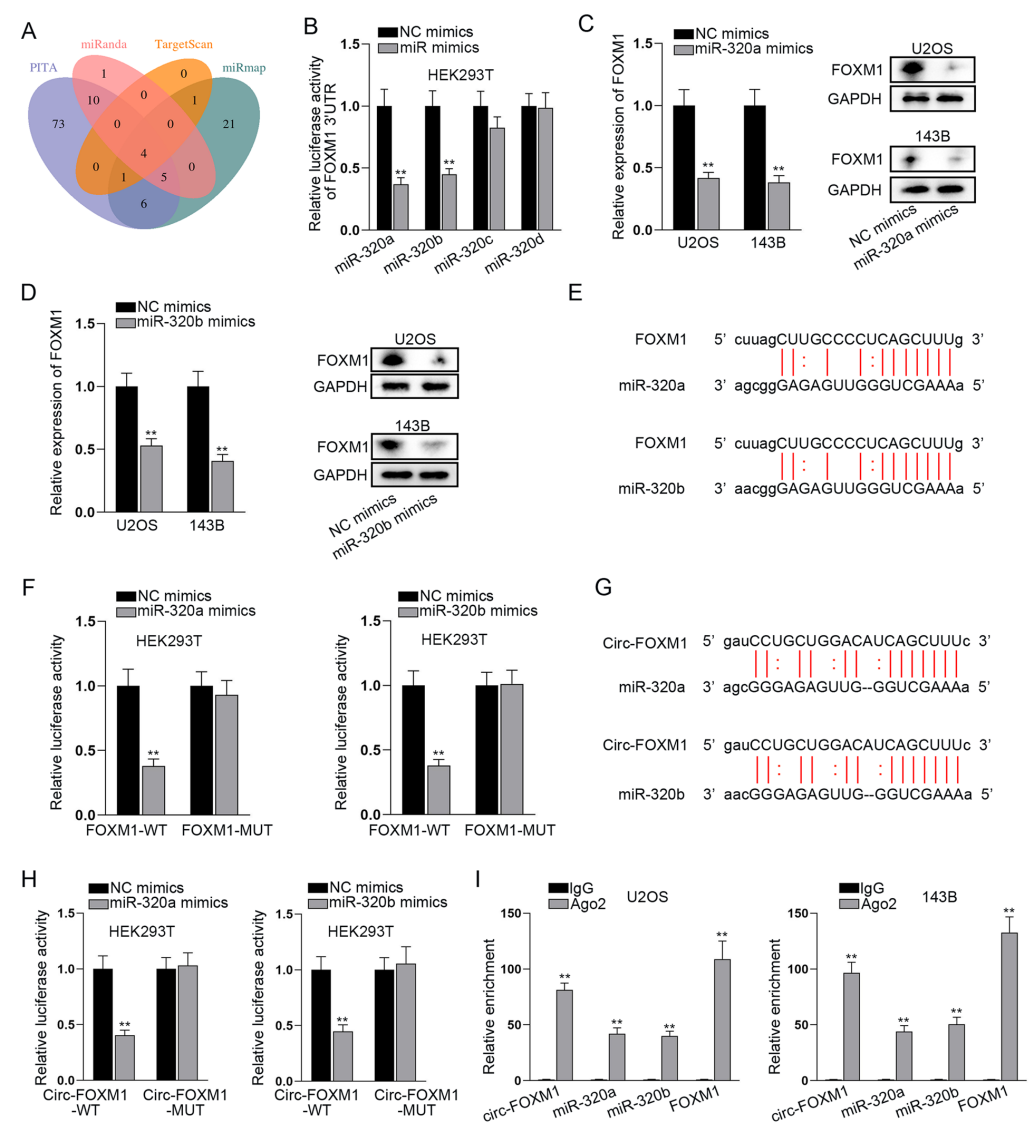
Circ-FOXM1 targeted miR-320a and miR-320b to modulate FOXM1. **a** The Venn diagram of miRNAs binding to FOXM1 from four databases (PITA, miRanda, TargetScan and miRmap) included in ENCORI website. **b** Luciferase reporter assay was utilized to screen out the miRNA targeting FOXM1. **c, d** The mRNA and protein levels of FOXM1 were detected through qRT-PCR and western blot when miR-320a and miR-320b were overexpressed in OS cells. **e** ENCORI predicted the binding sites between miR-320a or miR-320b and FOXM1. **f** Luciferase reporter assay was applied for proving the binding site of miR-320a or miR-320b and FOXM1. **g** ENCORI predicted the binding sites between miR-320a or miR-320b and circ-FOXM1. **h** Luciferase reporter assay was applied for proving the binding site of miR-320a or miR-320b and circ-FOXM1. **i** RIP assay tested the enrichment of miR-320a, miR-320b, circ-FOXM1 and FOXM1 in Ago2-RISCs. **P < 0.01

By ceRNA network, long ncRNAs (lncRNAs) or circRNAs may act as miRNA sponges to liberate miRNAs-targeted mRNAs [27, 28]. In order to explore the regulation mechanism of circ-FOXM1 on FOXM1 expression, we initially searched for the candidate miRNAs targeting FOXM1 by the utilization of Encyclopedia of RNA Interactomes (ENCORI, https://rna.sysu.edu.cn/encori/). Through the prediction of PITA, miRanda, TargetScan and miRmap algorithms in ENCORI, we obtained four putative miRNAs binding to FOXM1, including miR-320a, miR-320b, miR-320c and miR-320d (Fig. 4a). For further screening, we overexpressed the candidate miRNAs (Additional file 1: Figure S1) and then carried out luciferase reporter assay. The results indicated that the luciferase activity of FOXM1 3’UTR was dramatically restrained by miR-320a or miR-320b upregulation, while that had no marked change after overexpression of either of rest two candidate miRNAs (Fig. 4b), suggesting that FOXM1 could bind with miR-320a and miR-320b. Next, we disclosed that the mRNA level and protein level of FOXM1 could be repressed by upregulation of miR-320a or miR-320b (Fig. 4c, d), which implied that miR-320a or miR-320b is negatively correlated with FOXM1 expression. Further, the binding site for FOXM1 and miR-320a or miR-320b was predicted by ENCORI (Fig. 4e). Following, luciferase reporter assay indicated that the luciferase activity of FOXM1-WT was largely weakened by the overexpression of miR-320a or miR-320b (Fig. 4f), which further proved the binding between FOXM1 and miR-320a or miR-320b. Then, we detected the binding situation of circ-FOXM1 and miR-320a or miR-320b since ENCORI also predicted the binding site of circ-FOXM1 and miR-320a or miR-320b (Fig. 4g). As expected, the luciferase activity of circ-FOXM1-WT was overtly inhibited via the upregulation of miR-320a or miR-320b, whereas that of circ-FOXM1-MUT not affected (Fig. 4h). Additionally, data of RIP assays testified that circ-FOXM1, FOXM1, miR-320a and miR-320b coexisted in Ago2-assembled RISCs (Fig. 4i). These results indicated that circ-FOXM1 sponged miR-320a and miR-320b to regulate FOXM1 expression.

### Circ-FOXM1 accelerated OS progression through upregulating FOXM1 expression to activate Wnt signaling pathway

**Fig. 5:**
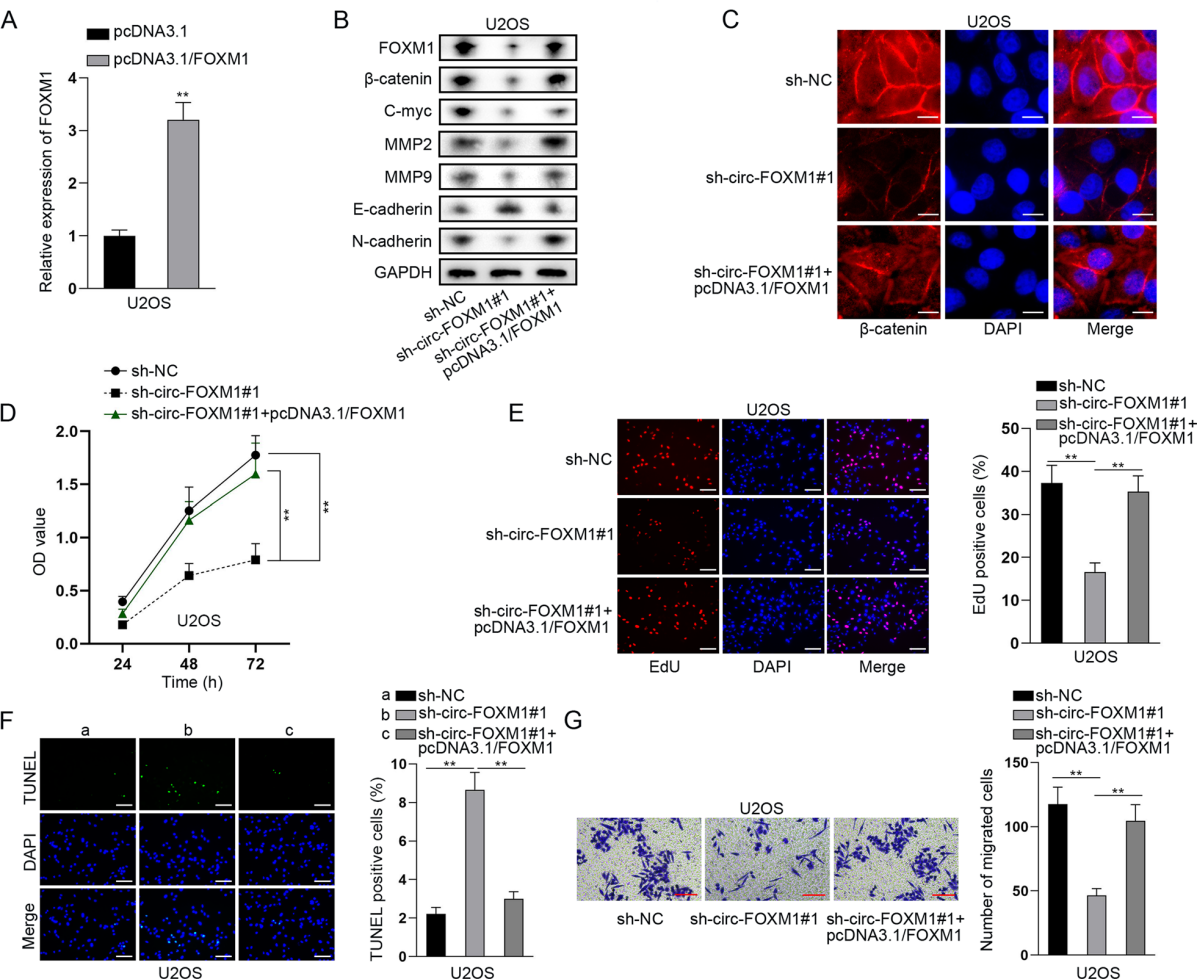
Circ-FOXM1 accelerated OS progression through upregulating FOXM1 to activate Wnt signaling pathway. **a** qRT-PCR analysis of the overexpression efficacy of FOXM1 in U2OS cells. **b** Western blot assay detected the level of FOXM1, β-catenin, C-myc, MMP2, MMP9, E-cadherin and N-cadherin in different groups. **c** IF (bar value = 20 μm) assay detected the level and distribution of β-catenin in different groups. **d, e** CCK-8 and EdU (bar value = 50 μm) assays evaluated the proliferation of OS cells in different groups. **f** TUNEL (bar value = 50 μm) assay measured the apoptosis of OS cells in different groups. **g** Transwell (bar value = 50 μm) assay examined cell migration in different groups. **P < 0.01

FOXM1 has been reported to promote β-catenin nuclear localization, thus activating Wnt signaling pathway and playing a tumor-promoting role in cancer [29]. Given that, we examined the regulatory role of circ-FOXM1/FOXM1 on Wnt signaling pathway. First of all, we overexpressed FOXM1 in U2OS cells by transfection of pcDNA3.1/FOXM1 (Fig. 5a). Then, we discovered that along with the changes in FOXM1 levels, the levels of key Wnt factor β-catenin and its downstream C-myc were significantly repressed by circ-FOXM1 knockdown and then this was totally reversed by overexpression of FOXM1. Similarly, the protein levels of MMP2, MMP9 and N-cadherin were also repressed by circ-FOXM1 inhibition and then completely reversed by FOXM1 upregulation, whereas that of E-cadherin exhibited opposite changes (Fig. 5b). Further, IF analysis results proved that the level of nuclear β-catenin was decreased by circ-FOXM1 knockdown but then increased by FOXM1 upregulation (Fig. 5c). These results indicated that circ-FOXM1 activates Wnt signaling pathway via upregulating FOXM1 expression. Then, rescue functional assays were further conducted to verify the regulatory mechanism of circ-FOXM1 and FOXM1 in OS cells. Through CCK-8 and EdU assays, we found that overexpression of FOXM1 could counteract the inhibitory effect of silenced circ-FOXM1 on OS cell proliferation (Fig. 5d, e). Besides, TUNEL assay illustrated that the increased cell apoptosis caused by the ablation of circ-FOXM1 could be reversed by FOXM1 upregulation (Fig. 5f). Transwell assay data showed that cell migration repressed by circ-FOXM1 deficiency could be recovered by overexpressing FOXM1 (Fig. 5g).

In addition, we conducted rescue experiments to detect the regulatory mechanism of circ-FOXM1, miR-320a and miR-320b in OS cells. In this regard, miR-320a inhibitor or miR-320b inhibitor was transfected into OS cells to block miR-320a or miR-320b, respectively. Unsurprisingly, we discovered that the inhibited cell viability and proliferation caused by circ-FOXM1 depletion could be partially rescued by miR-320a inhibitor alone but totally rescued by both miR-320a inhibitor and miR-320b inhibitor (Additional file 2: Figure S2A–B). Likewise, circ-FOXM1 knockdown elevated cell apoptosis was partially recovered by co-transfection of miR-320a inhibitor, while was fully reversed by co-transfection of miR-320a inhibitor and miR-320b inhibitor (Additional file 2: Figure S2C). Additionally, transwell assay data proved that the suppression impact of circ-FOXM1 down-regulation on OS cell migration was partly offset by miR-320a inhibition but completely countervailed by miR-320a/b inhibition (Additional file 2: Figure S2D). Further, western blot data proved that EMT process of OS cells and the activity of Wnt pathway repressed by circ-FOXM1 depletion could be partially rescued by miR-320a inhibitor alone but totally rescued by both inhibition of miR-320a and miR-320b (Additional file 2: Figure S2E). Altogether, circ-FOXM1 activated Wnt pathway to aggravate the malignant phenotypes of OS cells via sequestering miR-320a/b to enhance its host gene FOXM1.

### Loss of circ-FOXM1 hindered in vivo growth of OS xenograft

**Fig. 6:**
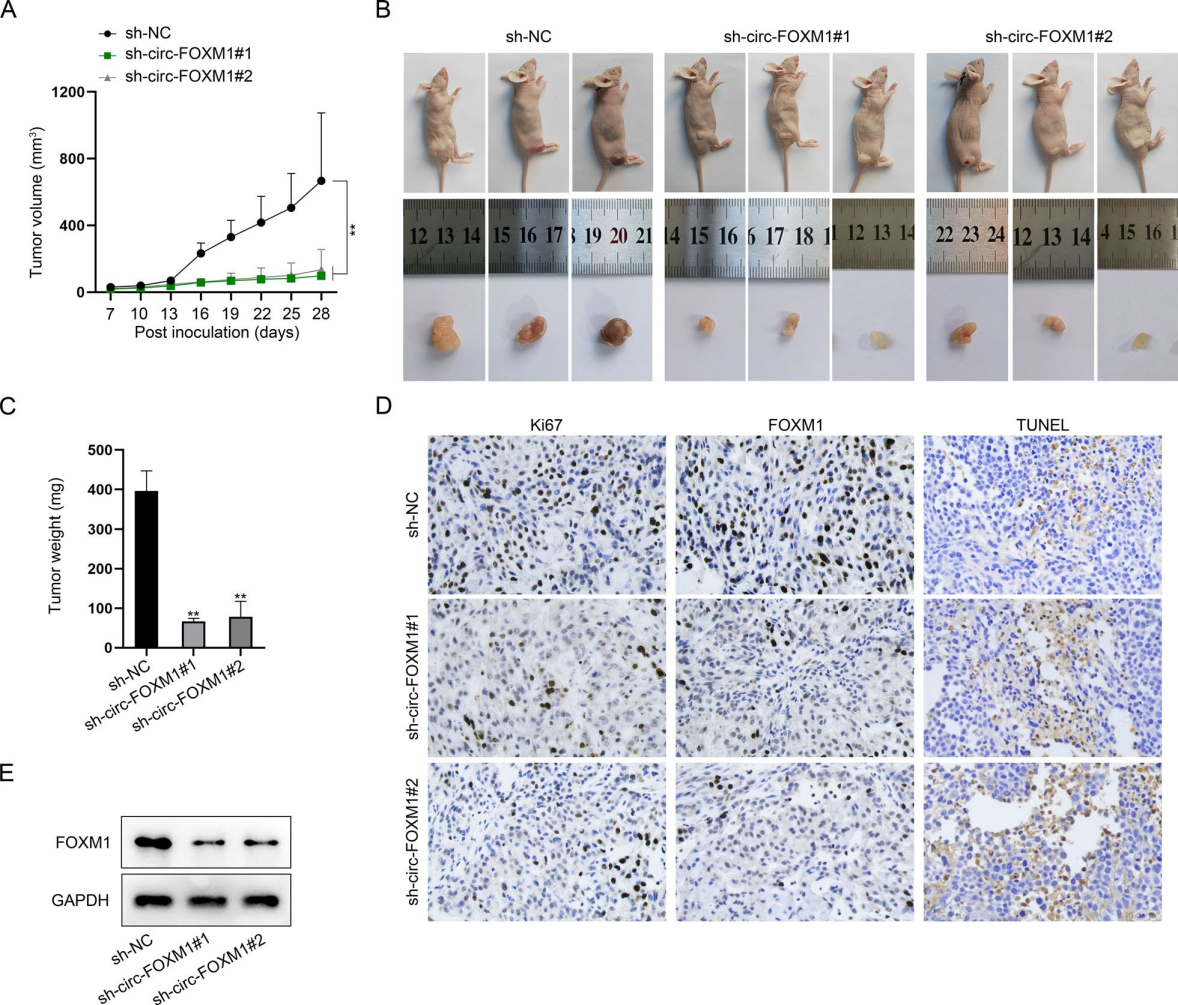
Loss of circ-FOXM1 restrained the growth of OS xenografts. **a** The growth curve of tumors from OS cells with or without circ-FOXM1 inhibition. **b** The images of tumor-bearing mice and xenografts from indicated groups. **c** Tumor weight in indicated groups. **d** IHC staining of Ki67 and FOXM1 as well as TUNEL staining in xenografts from indicated groups. **e** Western blot assessed FOXM1 level in xenografted tissues. **P < 0.01

In the end, we were interested in the significance of circ-FOXM1 in OS tumorigenesis. Hence, the in vivo tumorigenesis experiments were implemented. As shown in Fig. 6a, the growth rate of xenografts generated from circ-FOXM1-silenced OS cells was much slowed when comparing to that of tumors from control groups. Resultantly, the final size of circ-FOXM1-interfered tumors was smaller and the weight was lighter than that of the control xenografts (Fig. 6b, c). In the meantime, it manifested that compared with the xenografts from control group, the staining levels of Ki67 and FOXM1 were lowered, while TUNEL positivity was strengthened in circ-FOXM1-inhibited tumors (Fig. 6d). Consistently, western blot also detected the decreased FOXM1 levels in circ-FOXM1-depleted tumors (Fig. 6e). In sum, circ-FOXM1 contributed to in vivo OS tumor growth.

**Fig. 7:**
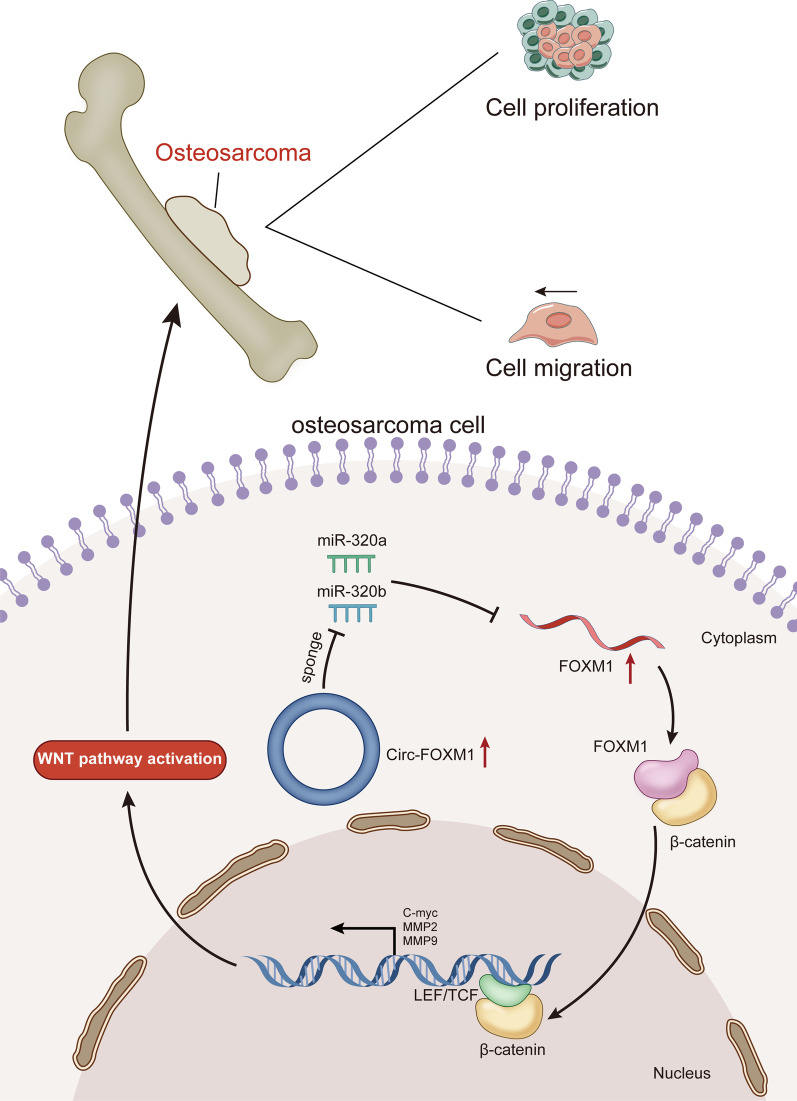
Graphical abstract. Circ-FOXM1 accelerated OS cell proliferation and migration through sponging miR-320a and miR-320b to upregulate FOXM1 expression and therefore activate Wnt signaling pathway

In conclusion, this research validated that circ-FOXM1 accelerated OS cell proliferation and migration through activation of the Wnt signaling pathway via sponging miR-320a and miR-320b to upregulate FOXM1 expression (Fig. 7).

## Discussion

Circular RNAs (circRNAs) are pivotal regulators for OS progression and therefore are proposed as promising biomarkers for the diagnosis, prognosis and treatment of OS patients [30]. For instances, a novel circulating hsa_circ_0081001 serves as a potential biomarker for the diagnosis and prognosis of patients with OS [31]. Down-regulated circ_HIPK3 suppresses the proliferation, migration and invasion of OS cells [32]. Circ-NT5C2 is an oncogene for OS cell proliferation and metastasis by targeting miR-448 [33]. Circ_0001721 indicates poor prognosis in OS, and it boosts OS cell progression through targeting miR-569 and miR-599 [34]. Although quite a few circRNAs including above ones have been reported to be related to OS development, the function and significance of many circRNAs have not been discussed in OS. Hence, it is meaningful to find as many circRNAs as possible that play a role in the progression of OS, so as to provide more potential targets for opening targeted drugs to treat OS.

Circ-FOXM1 (hsa_circ_0025033) is a circRNA generated from its host gene FOXM1, a reported oncogene and prognostic predictor for OS [35]. Formerly, circ-FOXM1 has been identified to exert the carcinogenic effects in several kinds of cancers such as glioblastoma [19], non-small cell lung cancer [20] and papillary thyroid cancer [21]. In this research, we affirmed that circ-FOXM1 expression was elevated in OS cells. In consistent with the conclusions from previous researches, this study, for the first time, displayed the oncogenic role of circ-FOXM1 in the proliferation and cell motility of OS cells. More importantly, in vivo data further identified the significance of circ-FOXM1 in OS tumorigenesis. Similar to above cited reports supporting other circRNAs as biomarkers for OS, our findings also provide preliminary evidences for circ-FOXM1 as a promising biomarker for OS. However, many other evidences are lost presently for circ-FOXM1 as an indeed biomarker for OS, such as clinical data and preclinical study.

Given that circRNAs usually have roles in cancers through regulating their host genes [36], here we explored the relationship of circ-FOXM1 and its host gene FOXM1 in OS. Past works have indicated the tumor-promoting role of FOXM1 in several cancers. For instance, miR-26b-targeted DEPDC1 facilitates cell proliferation via upregulating FOXM1 in TNBC [37]. FOXM1 deubiquitinated by USP21 modulates cell cycle and paclitaxel sensitivity of basal-like breast cancer cells [38]. Repression of Wnt3a/FOXM1/β-Catenin pathway is widely involved in the apoptotic impact of moracin D in breast cancer [39]. Consistent with the report of Zhu et al. [18], we verified that FOXM1 was expressed at high levels in OS cells. Further, we investigated the regulation of circ-FOXM1 on FOXM1 and found that circ-FOXM1 could positively regulate FOXM1 mRNA expression at post-transcriptional level but not at transcriptional level. Our study firstly uncovered the regulatory function of circ-FOXM1 on its host gene FOXM1 in OS.

Moreover, we investigated the regulatory mechanism of circ-FOXM1 on FOXM1 in OS. The participation of circRNAs in ceRNA network is universally known as one of their main post-transcriptional regulations on target mRNAs [40–42]. The ceRNA mechanism refers to that circRNAs can act as a ceRNA to sponge miRNAs, so as to regulate the expression of mRNAs [27]. Herein, we proved that circ-FOXM1 sequestered miR-320a and miR-320b to elevate FOXM1 expression in OS cells. Functionally, miR-320a and miR-320b are recognized as tumor suppressors in carcinomas, OS included. For example, miR-320 family is reported to be down-regulated in colorectal adenoma and repress cell proliferation by targeting CDK6 [43]. MiR-320a can inhibit cell growth and chemosensitivity through regulating ADAM10 in gastric cancer [44]. MiR-320b suppresses pancreatic cancer cell proliferation through regulating FOXM1 [45]. Importantly, reports have proven that miR-320a and miR-320b function as a tumor inhibitor in OS [46, 47], which is consistent with our research. In short, mechanism assays showed that circ-FOXM1 acted as a sponge of miR-320a and miR-320b to strengthen FOXM1 expression in OS cells. Similarly, a recent report also unveiled that circ-FOXM1 serves as a ceRNA of FOXM1 to modulate paclitaxel resistance of ovarian cancer cells [48].

## Conclusions

Taken together, our research demonstrated that circ-FOXM1 worked as a tumor promoter in OS via relieving the inhibition of miR-320a and miR-320b on FOXM1 expression, therefore activating Wnt pathway. All these findings presented the regulation mechanism of circ-FOXM1 in OS, offering a potential target for the treatment of OS patients.

## Supplementary Information

**Additional file 1: Figure S1**. The mimic efficiency of miR-320a/b/c/d. (A-D) The mimic efficiency of miR-320a/b/c/d was detected by qRT-PCR in OS cells transfected with mimics for miR-320a, miR-320b, miR-320c, or miR-329d, respectively. **P < 0.01

**Additional file 2: Figure S2**. Circ-FOXM1 accelerated OS progression through sponging both miR-320a and miR-320b. (A-B) CCK-8 and EdU (bar value = 50 μm) assays estimated OS cell proliferation in different groups. (C) TUNEL (bar value = 50 μm) assay detected OS cell apoptosis in different groups. (D) Transwell (bar value = 50 μm) assay examined OS cell migration in different groups. (E) Western blot evaluated the levels of FOXM1 and proteins related to Wnt pathway and EMT process in different groups. * P < 0.05, **P < 0.01

## Data Availability

Not applicable.

## Abbreviations

FOXM1: Forkhead box M1
circRNAs: Circular RNAs
ncRNAs: Noncoding RNAs
mRNAs: Messenger RNAs
miRNAs: MicroRNAs
ceRNA: Competing endogenous RNA
FBS: Fetal bovine serum
qRT-PCR: Quantitative real-time polymerase chain reaction
CCK-8: Cell counting kit-8
EdU: 5-Ethynyl-2'-deoxyuridine
DAPI: 4',6-Diamidino-2-phenylindole
TdT: Terminal deoxynucleotidyl transferase
TUNEL: DUTP nick-end labeling
RIP: RNA immunoprecipitation
RIPA: Radio immunoprecipitation assay
SDS-PAGE: Sodium dodecyl sulfate–polyacrylamide gel electrophoresis
WT: Wild type
Mut: Mutant
SD: Standard deviations
