# Supplementary figures and images for: Circ-FOXM1 promotes the proliferation, migration and EMT process of osteosarcoma cells through FOXM1-mediated Wnt pathway activation
Source: J Orthop Surg Res. 2022 Jul 7;17:344. doi: 10.1186/s13018-022-03207-0 (PMC9261067; doi:10.1186/s13018-022-03207-0)

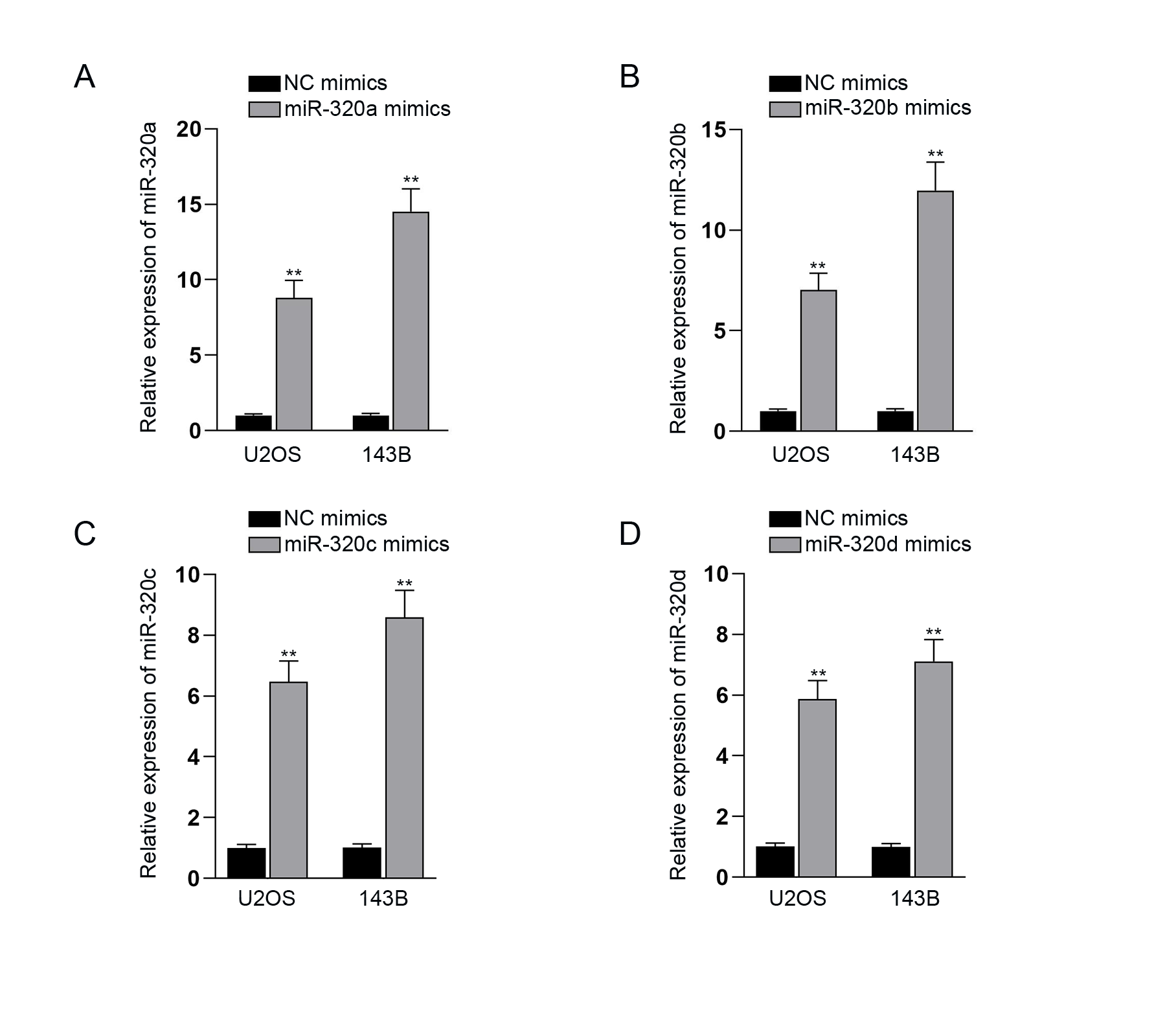

Supplement: Supplementary file 1 — Additional file 1: Figure S1. The mimic efficiency of miR-320a/b/c/d. (A-D) The mimic efficiency of miR-320a/b/c/d was detected by qRT-PCR in OS cells transfected with mimics for miR-320a, miR-320b, miR-320c, or miR-329d, respectively. **P < 0.01 [file 13018_2022_3207_MOESM1_ESM.tif]

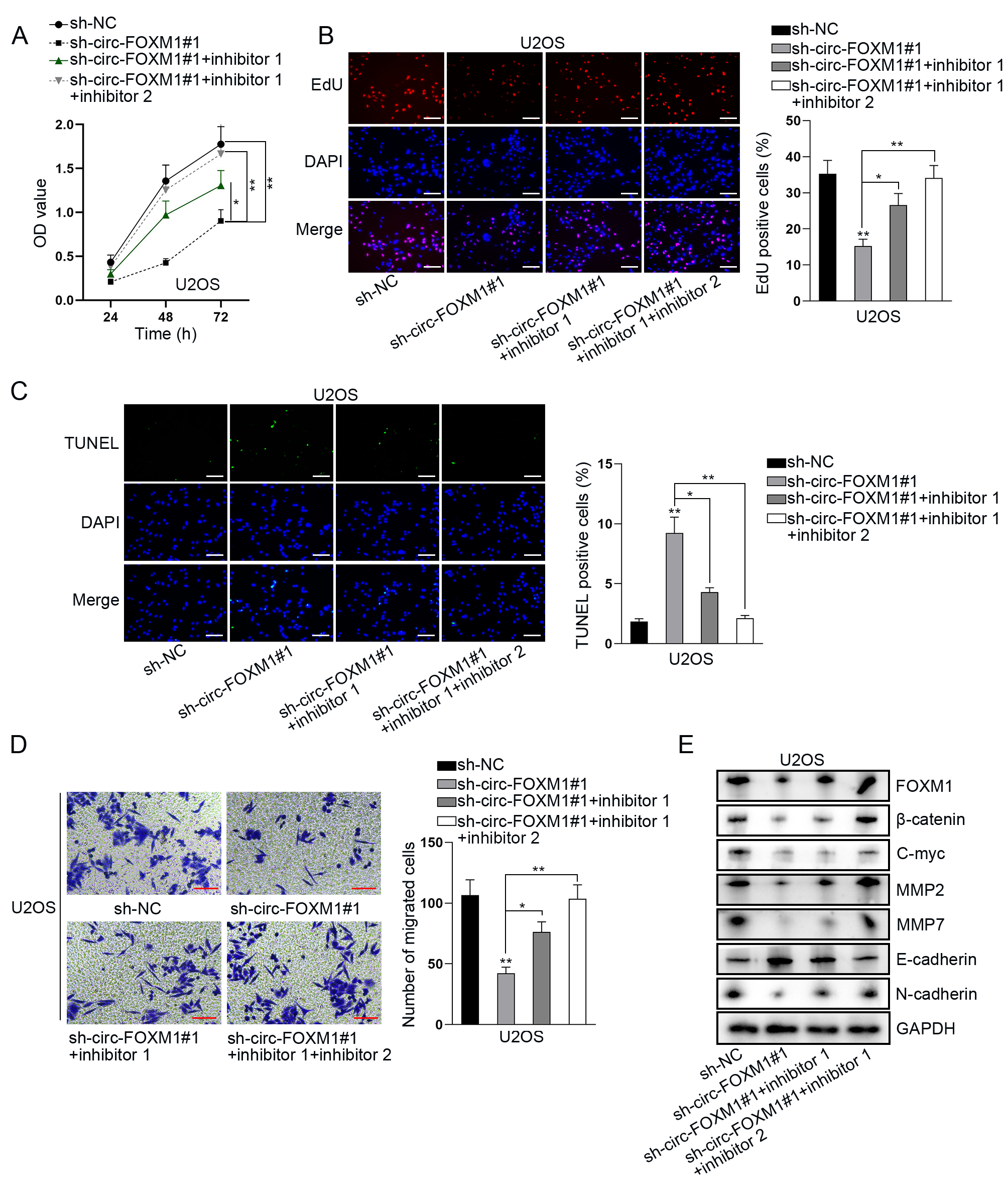

Supplement: Supplementary file 2 — Additional file 2: Figure S2. Circ-FOXM1 accelerated OS progression through sponging both miR-320a and miR-320b. (A-B) CCK-8 and EdU (bar value = 50 μm) assays estimated OS cell proliferation in different groups. (C) TUNEL (bar value = 50 μm) assay detected OS cell apoptosis in different groups. (D) Transwell (bar value = 50 μm) assay examined OS cell migration in different groups. (E) Western blot evaluated the levels of FOXM1 and proteins related to Wnt pathway and EMT process in different groups. * P < 0.05, **P < 0.01 [file 13018_2022_3207_MOESM2_ESM.tif]
